# From co‐design to co‐production: Approaches, enablers, and constraints in developing a public health, capacity‐building solution

**DOI:** 10.1111/ajr.12930

**Published:** 2022-10-17

**Authors:** Catherine Cosgrave, Alison Kennedy, Timo Dietrich, Kate Gunn, Joanna MacDonald, Claire McKay, Sally Cunningham, Sam Haren, Josh Lewis

**Affiliations:** ^1^ School of Rural Medicine University of New England Armidale New South Wales Australia; ^2^ National Centre for Farmer Health Deakin University Hamilton Victoria Australia; ^3^ Social Marketing @ Griffith, Griffith Business School Griffith University Brisbane Queensland Australia; ^4^ Department of Rural Health, Allied Health and Human Performance University of South Australia Adelaide South Australia Australia; ^5^ Sandpit Kent Town South Australia Australia; ^6^ Here! Here! Design Parkside South Australia Australia

**Keywords:** implementing evidence, new models and frameworks, public health, rural issues, rural mental health

## Abstract

**Introduction:**

Investigating how co‐designed knowledge can be translated to co‐produce a public health capacity‐building solution for difficult‐to‐engage population groups drawing on the co‐production experience of a prevention‐focused, capacity‐building mental health solution targeting primary producers.

**Design:**

A qualitative study undertaken in rural and regional Victoria involving members of the design working group including project team (7px), digital design team (5px), marketing team (3px), and funding partner representatives. The study design involved reflective practice to collect data to identify the phases of co‐production and assess the design working group members' experiences. The analysis involved inductive coding using Braun and Clarke's thematic analysis.

**Objective:**

Identifying major points of divergence and/or convergence; enablers and/or constraints; and ways to better navigate and strengthen the co‐production process.

**Finding:**

Given members of the design working group, diverse skills sets divergence was experienced in all co‐production phases. Divergence was also experienced between the project team and the funding partner given the uniqueness of working conditions and requirements of workers in the primary production industry. The project team applied an iterative development process to project management; encouraging iterative cycles to create/test/revise among the teams, and with the funding partner, until each was satisfied with the end result (convergence).

**Discussion & Conclusion:**

When developing a co‐created public health prevention campaign it is critical that the project team focuses on relationship building among the members of the design working group and ensures adequate resourcing, development of shared understanding of project goals and target audience, ongoing communication, and a commitment to working iteratively.

**What is already known on this subject:**

- Primary producers can be difficult to engage in public health and mental health prevention programs
- Co‐designing interventions can improve uptake, engagement, and ownership in difficult‐to‐reach populations
- Translating co‐designed knowledge into the co‐production of capacity‐building, online public health solutions can be challenging, due to the divergent skills sets, motivations, and professional languages of those involved

**What this paper adds:**

- Discusses how co‐designed design principles were translated and co‐produced into a prevention‐focused, capacity‐building mental health solution
- Identifies the enablers and constraints that a multidisciplinary team experienced during the co‐production process
- Offers strategies for successful collaboration when developing a co‐created public health prevention campaign

## 1 INTRODUCTION

Farmers and fishers (hereafter termed primary producers) commonly work in environments involving high levels of uncertainty and low levels of job control, placing them at considerable risk of poor‐mental health.^1^, ^2^, ^3^, ^4^ While primary producers are clearly in need of preventative mental health programs,^2^ they have been traditionally perceived as difficult to engage.^5^ Evidence suggests that increasing the acceptability of mental health prevention programs improves uptake by primary producers.^6^ To develop an ‘acceptable’ mental health prevention strategy requires a co‐designed approach with key stakeholders that have vested interests in the solution design. This article discusses a co‐designed^7^ intervention to prevent work‐related risks to mental health in primary production—the Primary Producer Knowledge Network (PPKN).

O'Reilly et al.^8^ highlight the issue of academics and experts falling into the trap of “conducting work on people or doing things to them” (p. 1). Ideating and designing with those you are seeking to serve is difficult. Similar issues and challenges have been observed in other research disciplines and are not uncommon.^9^ Calls for co‐creation that involves end‐users are made frequently, but beyond end‐users we must also draw on the collective knowledge of vested stakeholders.^10^ Co‐production literature highlights^11^ the need for equitable and experientially informed research which ensures that equitable partnerships “are formed by explicitly addressing inequalities in power so that they can actively contribute to, influence, and even direct the research process” (p. 4).^8^ Depending on the stakeholder group, we need to carefully explore who can and wants to contribute, to what extent, and when.^10^ Rundle‐Thiele et al.^12^ position co‐creation as an umbrella term (similar to Sanders and Stappers^13^) and then suggest a number of principles that require the application to ensure authentic and collective solution co‐creation. Within co‐creation the authors suggest that you may select from different methods and approaches that are most relevant for your particular aims. Examples of methods and processes are co‐design, co‐production, design thinking, the living lab, and persona creation.^12^ Our conceptualisation of co‐design and co‐production has evolved following the application of the 7‐step co‐design process in different behaviour change contexts covering environmental, health and social issues.^7^, ^14^, ^15^, ^16^

The purpose of a co‐design methodology is to provide a structure to facilitate input from the group of affected people into the development of the proposed ‘solution’, so that it is user‐centred and stakeholder supported.^9^ Co‐design is an incremental process, commonly starting with sourcing and reviewing relevant evidence to strengthen understanding and define the problem, before collecting information from end‐users and other stakeholders to understand preferences and needs, and gathering ideas for the creation of solutions.^17^, ^18^ The data gathered is then collated and analysed and can be used to establish key design principles to guide the development of the prototype that reflects the solution(s) that were ideated.^17^, ^18^ The development of the ‘solution’ was labelled as “building for change” by Trischler and colleagues^9^—the final stage of a seven‐step co‐design process (applied by the PPKN^7^). Here, we believe, exists further necessity to detail how the actual development process between researchers, external parties, experts, and end‐users is executed. “Building for change” draws on co‐design insights to generate a service solution by converting ideas into a prototype.^19^ The protype is then tested with the end‐users (and other key stakeholders) to gauge acceptability and level of support.^17^ This final stage of the co‐design process has been under‐explored to date and warrants more research attention.^19^ Contrary to other co‐production conceptualisations,^11^ we see co‐production as the next sequential step in the co‐design process which is focused on the implementation and testing of all developed ideas. It involves additional partners to build the service design solution which “were traditionally seen as the sole responsibility of professionals” (p. 476).^4^ Co‐production ideally involves rapid iteration cycles, overseen by a ‘design working group’ comprising different members or teams with specific expertise, along with end‐users as required.^17^, ^18^

In light of the above, this article aims to detail the experience of the co‐production process and document how the PPKN design working group undertook the solution build for the Campfire project. Details regarding the co‐design process and its outcomes have been published previously.^7^ This article identifies the enablers and constraints that the multidisciplinary PPKN design working group experienced during the co‐production process. The paper is guided by the following research question: *How can co‐designed knowledge be translated to co‐produce a public health capacity‐building solution for difficult to engage population groups?*

## 2 METHODS

### 2.1 Context

The PPKN commenced in April 2020 and concluded in January 2023, led by Australia's National Centre for Farmer Health and funded by the WorkSafe Victoria's WorkWell Mental Health Improvement Fund. The aim of the PPKN is to prevent work‐related risks to mental health among primary producers. To realise this aim, in June 2020 the PPKN project team commenced a co‐design process with primary producers and industry stakeholders, applying the seven‐step framework outlined by Trischler et al.^9^

A paper detailing the PPKN and the application of Trischler et al.'s^6^ co‐design framework was previously published.^7^ The article reflected on the online multi‐workshop process undertaken and proposed adaptions to Trischler et al.'s model when co‐designing online.^7^ Nine design principles were identified as an outcome of the co‐design process. These design principles were: (1) Personal connection is essential; (2) Keeping an eye on the goal; (3) Language matters; (4) One size will not fit all; (5) There is limited downtime as a primary producer; (6) Local matters; (7) Personalisation encourages engagement; (8) Virtual digital connection is becoming more acceptable and accessible in a range of formats; and (9) Utilise existing knowledge, resources and networks.^7^

**FIGURE 1:**
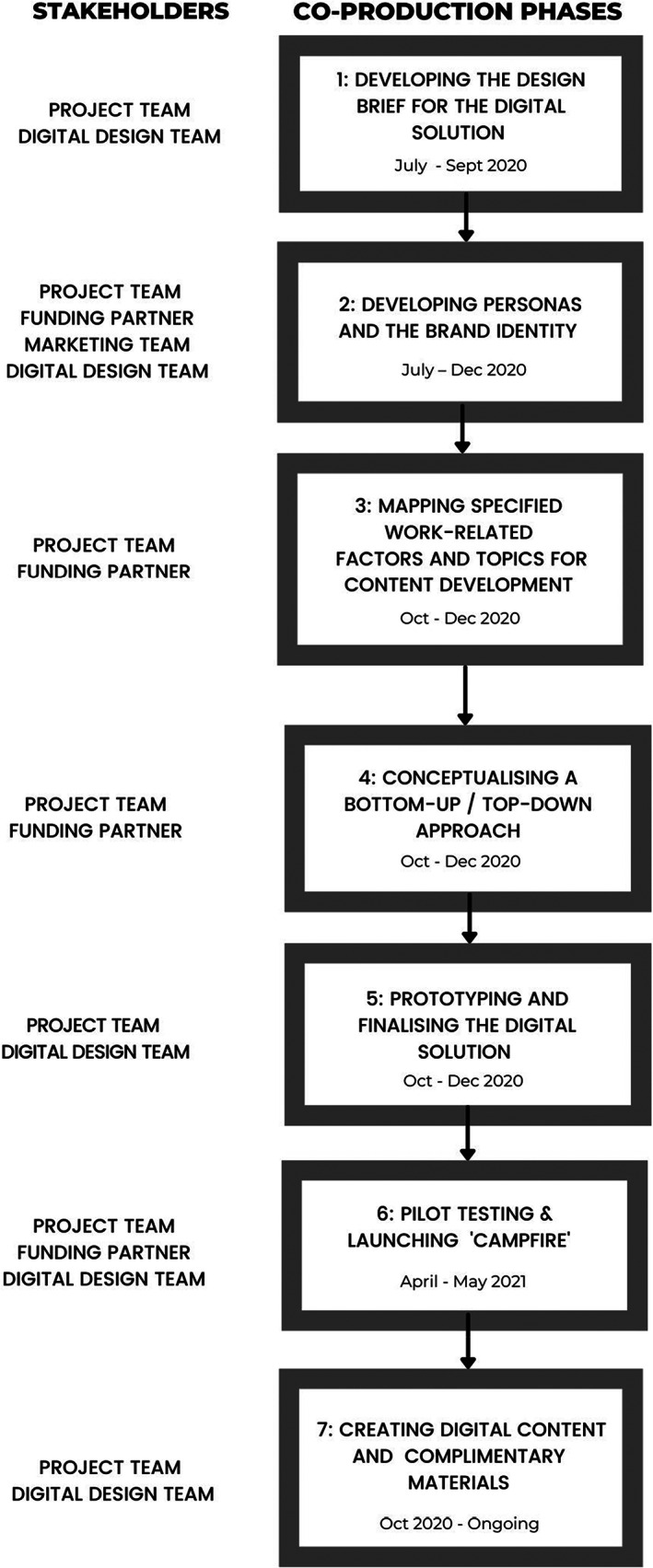
PPKN co‐production phases.

Following the completion of the co‐production process, the PPKN project team retrospectively reflected on the process undertaken. Seven overlapping phases were identified (see Figure 1): (1) Developing the design brief for the digital solution; (2) Developing personas and the brand identity; (3) Mapping specified work‐related factors and topics for content development; (4) Conceptualising a bottom‐up/top‐down approach; (5) Prototyping and finalising the digital solution; (6) Pilot testing and launching ‘Campfire’; and (7) Creating digital content and complementary materials.

### 2.2 The PPKN design working group members

The PPKN design working group involved multiple teams working together in different combinations across the identified phases (see Figure 1). These teams included: a project team, digital design team, marketing team, and the funding partner.

The project team is an interdisciplinary and cross‐sectoral team and includes six academics (five of whom are authors of this article [Cosgrave, Kennedy, Dietrich, Gunn and MacDonald]). Two research assistants joined the team during the co‐production process (Author 6 and Author 7). All the project team members have a focus on translational research, with experience using community‐based participatory research approaches including co‐design. The digital design team has expertise in physical design, digital media and strategy and the team included five members including the co‐author (Author 8). The marketing team has expertise in graphic design, web design, marketing, advertising and illustration and comprises three members including the co‐author (Author 9). The project funding partner was WorkSafe Victoria's WorkWell Mental Health Improvement Fund—established to support workplace leaders to prevent mental injury and promote safe and mentally healthy workplaces. WorkWell recognised the need for more support in agriculture and worked with PPKN to ensure there was a project focused on the agriculture industry.

### 2.3 Methods

After identifying the seven co‐production phases, the project team split into sub‐groups (2–3 people) and allocated phases of the project in which they were closely involved. Sub‐groups identified the key activities undertaken, based on recall and project documentation (e.g., reports, presentations, emails, and meeting notes). Author 1 then compiled narrative summaries of each phase which were sent to the co‐authors to check for accuracy. Co‐authors were then asked to assess their experience of participating in the PPKN design working group during each phase in which they were involved. Specifically, they were asked to: (1) Describe any major points of divergence (a drawing apart) and/or convergence (a coming together) experienced between the different teams; (2) Identify important enablers and/or constraints; and (3) Offer suggestions for better navigating and strengthening the co‐production process. Author 1 then collated the responses and thematically analysed them using Braun and Clark's inductive coding approach.^20^ The responses and themes were checked by Author 2 for validity and appropriateness. A final draft of the article was sent to all co‐authors and the funding partner to check and approve the interpretations made.

### 2.4 Ethical considerations

The PPKN project was approved by Deakin University's Faculty of Health Ethics Committee (HEAG‐H79_2020)

## 3 RESULTS

A description of the activities undertaken, and the experiences of the participating teams are included under each phase:

### 3.1 Developing the design brief for the digital solution

The co‐production process commenced in June 2020 with the project team delivering the ‘co‐design report’ to the digital design team (co‐design details are published in Kennedy et al.^7^). The report included the concept stage user‐driven solutions and nine design principles (reported in methods section). After the delivery of the co‐design report, both teams met regularly to develop the design brief for the digital solution. The aim was for the design team to develop an understanding of the context, research, and findings from the co‐design process.

The design team felt they started with a good understanding of the primary production sector and awareness of the diversity of potential end‐users given previous involvement with the National Centre for Farmer Health. The co‐design report and discussions with the project team supported the development of a shared understanding of the project context. However, divergence between the teams existed around different interpretations and prior experience with co‐design processes and frameworks. For example, the digital design team was unfamiliar with the use of activity cards to generate ideas during co‐design. Initially viewing these as frontloading participants with a pre‐determined solution, the digital design team later recognised their purpose of supporting and empowering further idea generation.

### 3.2 Developing personas and the brand identity

At the request of the digital design team, four personas were developed to reflect a range of primary producer identities—with the purpose of assisting convergence around an understanding of the design solution's target audience. A persona's purpose is to create a representation of the target audience. Each persona comprised an in‐depth personal profile featuring a visual representation and short biography of demographic, geographic, psychographic and behavioural information. Persona creation was an iterative process and included the project team drawing on their contextual expertise and feedback from the funding partner. For the digital design team, the personas become emblematic of the target primary producer audience, helping to guide the solution build. The personas also helped the funding partner identify divergence over the priority of target audiences; with the project team being focussed on family farming business structures, while the funding partner identified a missing focus on primary producer employers with a significant external workforce. It also re‐emphasised the funding partner's focus on work‐related factors which are defined as factors in the design or management of work that can put an employee's mental health at risk. The inclusion of work‐related factors resulted in the addition of a fifth persona and the inclusion of relevant work‐related factors for each persona.

A brand identity and essence statement were developed with the aim to embed, focus and articulate the PPKN's project goals, objectives, language and values, as well as differentiate the PPKN's design solution from other mental health interventions. This was also an iterative process, involving input from the entire PPKN design working group. The final agreed name and logo was ‘Campfire’.

The original funding proposal did not allocate resourcing for the creation of personas or a brand narrative or include the preparation of presentations on these concepts to various stakeholders (time‐intensive activities). However, the personas and the brand narrative served as important elements that helped to drive convergence between all teams as they reminded everyone who the target audience was and what the purpose, objective and values of Campfire were.

### 3.3 Mapping specified work‐related factors and identifying topics areas for content development

The digital solution was developed to directly address work‐related factors on primary producers' mental health (low job control, poor support, poor‐environmental conditions, high and low job demands, poor organisational change management, low role clarity, poor‐workplace relationships, remote and isolated work, low recognition and reward, and violent and traumatic events). Work‐related factors relevant to primary producers were mapped against the information gathered from the literature, expert interviews and the co‐design findings,^7^ an approach that is consistent with protocols for developing effective behaviour change interventions.^20^ This process helped the project team convey to the digital design team important behavioural targets that the digital solution needed to address, and helped to communicate to the funding partner the linkages (perceived by the project team) between the evidence collected to date and the work‐related factors. The process also informed the later identification and development of targeted, contextually appropriate topics for inclusion on the resulting Campfire platform (see phase 7 for example topics identified).

Divergence was noted at points in the process of solution development, due to differences in expectations towards the delivery and communication of a WorkWell funded project. To navigate and negotiate the divergence, the project team worked closely with the funding partner and related stakeholders to gain better understanding of work‐related factors. The language was adjusted in, and re‐alignment to the work‐related factors occurred with, the elements of the Campfire topics. This resulted in a solution that closely aligned with and contributed to the evidence base of the broader WorkWell program, while remaining a focused and targeted intervention for primary producers.

### 3.4 Conceptualising a bottom‐up/ top‐down approach

The project team recognised a tailored solution was needed to engage the target audience, particularly given the multitude of factors that differentiated primary production from other workplaces (e.g., frequent family business structures, co‐location of work and home, seasonal workforce, and multi‐generational workforce). The unique nature of primary production contexts and distinctive requirements of primary producers, therefore, posed challenges to the project team in terms of aligning with the overall goals and approaches of the broader WorkWell program. As such, the project required re‐negotiation and re‐alignment of approaches to tailor solutions for primary producers, including the adaption of language, the inclusion of relatable peer stories, and a focus on expert‐informed practical strategies and solutions. The end result was able to reconcile the divergence and create intervention solutions that drew on the existing evidence base to prevent and manage work‐related risk factors specific to primary production contexts.

### 3.5 Prototyping and finalising the digital solution

The digital design team—using the design brief (phase 1), along with the knowledge gained from phases 2, 3, and 4—developed a digital prototype. This prototype comprised a web‐based, text‐driven platform modelled on a campfire conversation and supported by the capacity to share and store images, links and resources. Following feedback and acceptance by the project team, the prototype was presented to the PPKN's Project Advisory Group (PAG) which included primary producers and industry representatives (many of whom had been involved in the co‐design process). A further online feedback workshop was held with a broader group of co‐design participants and industry stakeholders to assess the acceptability and face validity of the Campfire platform. As part of finalising the Campfire platform, the digital design team developed a list of potential features and asked the project team to rank their priority as either ‘must’, ‘could’, ‘should’ or ‘do not’ include (balancing cost and usability).

Divergence was experienced between the project team and the digital design team during this phase. While the project team was concerned with creating a digital design solution that met the project goals and drove end‐user engagement; the digital design team was also focused on delivering a novel solution with a creative aesthetically designed user experience. While an acceptable compromise was reached that met the project goals, the resulting level of complexity extended the project timelines and required some adjustment to practical considerations of the digital solution (e.g., layout and functionality of the digital interface). Further adjustments were made after the launch of the digital platform (see Phase 6).

### 3.6 Pilot testing and launching ‘Campfire’

The Campfire platform had two phases of pilot testing (alpha and beta). Alpha testing was conducted by the project team and initial feedback regarding faults and issues were provided to the digital design team. After addressing the initial feedback, Campfire underwent beta testing involving primary producers and industry stakeholders drawn from the earlier co‐design workshops. A broader call for community stakeholders increased the beta testing group. Participants involved in beta testing were provided with one of the personas (developed in phase 2), with instructions to reflect this target audience and complete specific tasks when testing the platform's functions and useability (e.g., bookmark a shared link, ask a question during a Campfire conversation, or attempt to use a banned swear word). The project team captured participants' feedback from the beta testing using a Google form which was then passed on to the digital design team to address. After changes were made (e.g., widening of the chat screen to improve visibility, updating user location options to a more familiar list of Victorian regions, and improving the visibility of the user's name in the chat box), a soft‐launch of Campfire was made to provide the opportunity for identifying and rectifying any previously unidentified errors (resulting in the further addition of ‘reply to’ message functionality).

The project team and digital design team experienced challenges during pilot testing. From the project team's side, the challenge was twice needing to identify, invite and coordinate a large group of test users and then collate their feedback under time constraints. From the digital design team's side, the challenges related to the novel platform design, requiring a more complex and extended testing process compared with more mainstream digital solutions (e.g., website).

### 3.7 Creating digital content and complementary materials

A sub‐group of the project team (Kennedy, McKay and Cunningham) were responsible for overseeing the creation of Campfire's digital content and other complementary resources such as a blog and podcast series and other promotional materials. Initially, the blogs and podcasts were created to help build the audience for Campfire's bonfire Q&A events (bonfires were facilitated online events held fortnightly with guest experts, to focus on topics identified as potentially creating work‐related risks to mental health [e.g., working effectively in a family business, communication—it's more than words, planning for success[ion], managing fatigue, and staying socially connected]). These blogs and podcasts were also found to be an effective way to engage primary producers not accessing the interactive digital platform. This addressed an identified need from the co‐design findings—while digital communication was increasingly acceptable to primary producers, non‐digital communication methods (such as face‐to‐face conversations [via Campfire roadshow events], hard copy resources, and podcasts and blogs) were also needed for those not wanting to engage digitally. Both the digital and complementary resources were developed iteratively between the project team sub‐group, the guest experts (including primary producers and topic experts), and the funding partner. This involved a process of feedback and review by the funding partner before finalisation and posting/publication. It also resulted in a hard‐copy brochure being developed to communicate the work‐related factors (and relevant practical solutions) to primary producers attending PPKN promotion events.

Some divergence between the funding partner and the project team emerged around the choice of language and the focus of blog and podcast content. To navigate this process, the project team worked to transfer knowledge regarding work‐related factors into simple language and practical messaging that was relevant and relatable to the primary production contexts. The tailoring of language surrounding work‐related factors was especially important, considering the importance of communication and tailored messaging for developing shared understandings of complex subject matters, which had been specified by primary producers in the co‐design sessions.

## 4 DISCUSSION

In the PPKN co‐production process, divergence between the PPKN design working group's teams was anticipated by the project team given the known differing expertise and skill sets involved, the planned use of creative digital technologies and the diverse group of end‐users. The PPKN was a large‐scale and ambitious project directed at the whole primary production sector (rather than starting out with a single sector, e.g., dairy farming). In response to these convergence risks, the project team applied an iterative development process to project management; encouraging iterative cycles of create, test, and revise among the teams until each was satisfied with the end result (convergence). An iterative process can be time consuming and resource intensive and should be factored into initial timelines and budgets for projects engaging with multiple stakeholders.

Despite being funded by WorkWell to implement learnings and contribute to the evidence base to improve general workplace mental health and wellbeing in Victoria, the uniqueness of working conditions and requirements of workers in the primary production industry meant that the expectations, approaches and objectives of the overall WorkWell program were not always applicable to the PPKN project. This created initial challenges in co‐designing and co‐production in alignment with WorkWell principles. Establishing a shared understanding of objectives between partners and stakeholders is vital to position a project to progress in a timely, focused and effective manner. This can be enabled by early‐stage project‐specific meetings (bringing together all stakeholders). Followed by regular contract management meetings to present stakeholder's knowledge of the issue, the target audiences and conceptual frameworks being used, and to identify and address any significant differences in expectations and understandings throughout the co‐design and co‐production processes. Should any divergence be identified, it is crucial for all partners involved to establish a process to iteratively work towards convergence through knowledge sharing, conflict resolution, and problem‐solving systems.

The project team needs to be conscious when contracting digital technology, marketing teams or other commercial partners for co‐production that the priorities, approach, and services required will differ from a co‐designed research/service delivery project (e.g., funding and governance obligations). These differences (and resulting expectations) need to be communicated and understood by all parties and clearly articulated in the tender or invitation to quote documents, where possible.

Involving commercial partners (e.g., digital design and marketing) in the early stages of co‐design would help build understanding of the context and the end‐users from the outset, creating a more time and budget‐efficient process of solution development. This would influence decisions relating to simplicity/complexity and the level of creativity needed for the digital solution.

### 4.1 Limitations

This article reports on the enablers and constraints experienced by the teams involved in the PPKN design working group. There may have been different things to consider if different teams (other than digital design, marketing, and funders) had been involved, e.g., public health services. The solution development (and the resulting interplay of stakeholders) was influenced by the COVID environment. Considerations may have differed in a non‐COVID environment where face‐to‐face engagement between the teams would likely have occurred.

## 5 CONCLUSION

While the quality improvement measures suggested in this article are not necessarily novel, they are no less important to reinforce. Relationship building, adequate resourcing (time, staffing, and budget), shared understanding of project goals and the target audience, ongoing communication, and a commitment to work iteratively to meet project objectives in spite of varying stakeholder priorities are critical when developing a co‐created public health prevention campaign.

## AUTHOR CONTRIBUTIONS

CC: conceptualization; data curation; formal analysis; investigation; methodology; writing – original draft; writing – review and editing. AK: conceptualization; data curation; formal analysis; investigation; methodology; writing – original draft; writing – review and editing. TD: conceptualization; formal analysis; investigation; writing – original draft; writing – review and editing. KMDG: conceptualization; formal analysis; investigation; writing – original draft; writing – review and editing. JM: writing – review and editing. CM: writing – review and editing. SC: writing – review and editing. SH: software; writing – review and editing. JL: resources; writing – review and editing.

## CONFLICT OF INTEREST

The authors declare no conflict of interest.

## DISCLOSURE STATEMENT

The Primary Producer Knowledge Network (PPKN) was funded by the WorkSafe Victoria's WorkWell Mental Health Improvement Fund.

## ETHICAL APPROVAL

The PPKN project was approved by Deakin University's Faculty of Health Ethics Committee (HEAG‐H79_2020).
